# Insights Into the Significance of the Chinense Loess Plateau for Preserving Biodiversity From the Phylogeography of *Speranskia tuberculata* (Euphorbiaceae)

**DOI:** 10.3389/fpls.2021.604251

**Published:** 2021-02-04

**Authors:** Jun-Wei Ye, Hai-Yang Wu, Meng-Jiao Fu, Pei Zhang, Bin Tian

**Affiliations:** ^1^Key Laboratory for Forest Resources Conservation and Utilization in the Southwest Mountains of China, Ministry of Education, Southwest Forestry University, Kunming, China; ^2^Germplasm Bank of Wild Species, Kunming Institute of Botany, Chinese Academy of Sciences, Kunming, China

**Keywords:** Chinese Loess Plateau, refugia, nuclear microsatellites, Quaternary, *Speranskia tuberculata*

## Abstract

The significance of the Chinese Loess Plateau (CLP) in maintaining biodiversity for northern China has rarely been shown, as previous phylogeographic studies are mostly woody species and they have revealed that Quaternary refugia are mainly located in mountain regions. We selected a drought-enduring endemic herb, *Speranskia tuberculata* (Euphorbiaceae), to determine its glacial refugia and postglacial demographic history. To this end, we sampled 423 individuals from 38 populations covering its entire geographic distribution. Three chloroplast DNA (cpDNA) fragments, two low-copy nuclear genes, and six nuclear microsatellites (nSSRs) were used and supplemented with ecological niche modeling (ENM) to infer the phylogeographic history of this species. Populations with private haplotypes and high haplotype diversity of cpDNA are mainly located in the CLP or scattered around northeastern China and the coastal region. Spatial expansion, detected using a neutrality test and mismatch distribution, may have resulted in a widely distributed ancestral cpDNA haplotype, especially outside of the CLP. For nuclear DNA, private haplotypes are also distributed mainly in the CLP. In nSSRs, STRUCTURE clustering identified two genetic clusters, which are distributed in the west (western cluster) and east (eastern cluster), respectively. Many populations belonged, with little to no admixture, to the western cluster while (hardly) pure populations of the eastern cluster were barely found. Genetic differentiation is significantly correlated with geographic distance, although genetic diversity is uniformly distributed. ENM suggests that the distribution of *S. tuberculata* has recently expanded northwards from the southern CLP, whereas it has experienced habitat loss in the south. Thus, *S. tuberculata* populations probably survived the last glacial maximum (LGM) in the southern CLP and experienced post-glacial expansion. Wind-dispersed pollen could bring the majority of genotypes to the front during spatial expansion, resulting in uniformly distributed genetic diversity. Based on evidence from molecular data and vegetation and climate changes since the LGM, we conclude that drought-enduring species, especially herbaceous species, are likely to have persisted in the CLP during the LGM and to have experienced expansion to other regions in northern China.

## Introduction

The Chinese Loess Plateau (CLP), which spans from 34 – 40°N and 101 – 113°E in northern China, contains heterogeneous topography and climate (Zhang et al., 2002) and is at the intersection of multiple floristic regions (Wang, 1999; Wu et al., 2010). A number of relict taxa and a diverse drought-enduring plants indicate that the CLP could have acted as a macrorefugium during Quaternary climate fluctuations (Harrison et al., 2001; Zhang et al., 2002; Ni et al., 2010). However, phylogeographic investigations of plants in northern China are limited (Qiu et al., 2011) as previous studies are focused on northwestern China (Meng et al., 2015), subtropical China (Ye et al., 2017a), northeastern China (Ye et al., 2017b) or southwestern China (Liu et al., 2017). Thus, the significance of the CLP for preserving the biodiversity of adjacent regions may be underestimated.

Paleovegetation reconstruction for northern China indicates that steppe and even desert vegetation covered this area during the last glacial maximum (LGM, ca. 26,000 – 18,000 years ago), while the present coniferous and deciduous forests experienced southward retreat to below 30°N (Harrison et al., 2001; Ni et al., 2010). Phylogeographic studies, however, challenged this hypothesis by discovering multiple LGM refugia, which have allowed species to persist across northern China (Chen et al., 2008; Tian et al., 2009; Zeng et al., 2011). In an endemic and dominant coniferous tree, *Pinus tabulaeformis*, Chen et al. (2008) suggested that there are multiple geographically isolated refugia based on restrictedly distributed mitotypes, although the plastid haplotypes were almost homogeneously distributed, which may be due to greater mobility of pollen relative to seed. Multiple localized refugia patterns were also discovered in a tree, *Quercus liaotungensis* (Zeng et al., 2011) and a shrub, *Ostryopsis davidiana* (Tian et al., 2009), as different plastid haplotypes were geographically restricted. Other studies on widely distributed East Asian species have shown that northern China is likely a contact zone between southern subtropical and northern cold temperate populations (Guo et al., 2014; Bai et al., 2016).

Previous studies have largely focused on woody plants (Chen et al., 2008; Tian et al., 2009; Zeng et al., 2011); therefore, these results may not be representative of the biome as a whole. Herbaceous species, especially drought-enduring plants that are mainly distributed in the CLP (Zhang et al., 2002) with much shorter generation times compared to woody species, may have responded differently to past climate changes (Wang et al., 2015). Second, only haplotypes of cytoplasmic markers are used to infer population demographic histories (Chen et al., 2008; Tian et al., 2009; Zeng et al., 2011), whereas the importance of pollen-mediated gene flow has been largely ignored except for *P. tabulaeformis* (Chen et al., 2008). As a result, many aspects of past population and range dynamics in northern China still need further exploration to provide insights into the preservation of biodiversity.

To assess the significance of the CLP for preserving biodiversity, *Speranskia tuberculata* (Bunge) Baillon (Euphorbiaceae), belonging to an East Asian endemic genus with two species only, was selected. This species is a drought-enduring endemic herb in northern China that is mainly distributed in the CLP and adjacent areas, where it usually occurs in dry habitats such as xerothermophilous grasslands, sparse thickets, and forest edges. Its pollen is dispersed by wind, while seeds are dispersed by gravity (personal observations). Its generation time is about one year base on personal observations. Genetic variation estimated from chloroplast (cp) and low-copy nuclear (n) DNA as well as nuclear microsatellites (nSSRs) was combined with ecological niche modeling (ENM) to investigate (a) the population genetic structure of *S. tuberculata* and to test (b) whether any refugia were located in the CLP.

## Materials and Methods

### Sampling and DNA Extraction

A total of 423 *S. tuberculata* individuals representing 38 populations were sampled through its entire geographic distribution (Table 1). We avoided collecting clones by sampling individuals more than 10 m apart. Genomic DNA was extracted from silica gel-dried leaves using the Ezup DNA Extraction Kit (Sangon Biotech, Shanghai, China). Voucher specimens were deposited in the herbarium SWFC (Southwest Forestry University), Kunming, China.

**TABLE 1:** Details of sample location, sample size, and genetic diversity of chloroplast DNA and nuclear microsatellites in 38 *Speranskia tuberculata* populations.

**Population**	**Location**	**Lat**	**Long**	**Ele (m)**	**cpDNA**				**Nuclear microsatellites**						
					**n**	***H*_d_**	**π (×10^–4^)**	**Haplotype (n)**	***n***	***A*_O_**	***H*_E_**	***H*_O_**	***R*_S_**	***P*_AR_**	***F*_is_**
AHQ	Aohanqi, Inner Mongolia	42.53	120.58	670	15	0.42	1.6	H1(11), H2(4)	15	28	0.57	0.56	2.97	0.00	0.06
BJ	Mentonggou, Beijing	39.98	116.03	400	10	0	0	H1(10)	10	28	0.54	0.43	3.29	0.07	0.25
CF	Wengniuteqi, Inner Mongolia	42.97	118.98	631	12	0	0	H1(12)	12	26	0.52	0.50	3.05	0.05	0.08
CFS	Chifeng, Inner Mongolia	42.42	118.92	1500	14	0	0	H1(14)	12	26	0.53	0.60	3.2	0.07	–0.08
DF	Dengfeng, Henan	34.52	112.93	650	16	0.49	3.3	H1(11), **H5(4), H6(1)**	16	22	0.45	0.42	2.6	0.02	0.11
DL	Dalian, Liaoning	38.92	121.62	130	10	0	0	**H26(10)**	–	–	–	–	–	–	–
FF	Fufeng, Shaanxi	34.28	107.90	530	8	0.71	4.9	H10(4)**, H9(2), H21(2)**	6	23	0.59	0.47	–	–	0.31
GG	Gangu, Gansu	34.82	105.30	1325	5	0	0	H1(5)	5	12	0.32	0.37	–	–	–0.02
GH	Guanghe, Gansu	35.50	103.57	2178	17	0	0	**H12(17)**	17	25	0.54	0.46	2.96	0.04	0.19
GS	Huixian, Henan	35.57	113.52	980	14	0.58	5.2	H1(5), **H15(1), H16(8)**	14	27	0.62	0.63	3.41	0.00	0.02
GY	Guyuan, Ningxia	36.27	106.40	1927	3	0	0	H1(3)	3	15	0.44	0.42	–	–	0.29*
HC	Hancheng,Shaanxi	35.40	110.38	470	4	0	0	**H8(4)**	4	17	0.46	0.38	–	–	0.32*
HM	Houma, Shanxi	35.58	111.40	530	8	0.43	1.6	H1(6), H7(2)	8	24	0.63	0.49	3.46	0.00	0.30*
HT	Huhehaote, Inner Mongolia	40.87	111.60	1147	14	0	0	H1(14)	14	23	0.54	0.61	2.94	0.00	–0.10
HY	Heyang, Shaanxi	35.02	110.22	475	9	0.22	0.9	**H20(8)**, H1(1)	9	19	0.52	0.59	2.7	0.00	–0.07
JB	Jingbian, Shaanxi	37.45	108.57	1689	1	0	0	H7(1)	–	–	–	–	–	–	–
JNS	Jinan, Shandong	36.62	117.07	250	14	0	0	H1(14)	14	21	0.55	0.39	2.74	0.03	0.32*
KLQ	Kulunqi, Inner Mongolia	42.75	121.38	400	8	0	0	H1(8)	8	25	0.59	0.69	3.26	0.08	–0.10
KQ	Keqi, Inner Mongolia	43.27	117.55	2067	13	0	0	H1(13)	13	21	0.47	0.39	2.76	0.09	0.22
LL	Luliang, Shanxi	37.12	111.15	1587	13	0	0	H7(13)	13	26	0.60	0.46	3.2	0.10	0.26
LX	Longxian, Gansu	34.92	104.68	1850	14	0.14	0.5	**H11(1)**, H7(13)	13	22	0.55	0.47	2.9	0.00	0.19
MZ	Mizhi, Shaanxi	37.78	110.12	1050	16	0.23	0.9	H7(14), **H17(2)**	16	29	0.65	0.76	3.37	0.00	–0.13
PC	Pucheng, Shaanxi	34.95	109.58	488	13	0	0	H1(13)	13	21	0.55	0.36	2.9	0.03	0.40
QD	Qingdao, Shandong	36.13	120.72	170	10	0	0	H1(10)	12	20	0.53	0.38	2.81	0.00	0.32
QY	Qingyang, Gansu	35.72	107.65	1408	11	0.18	0.7	H1(10), H7(1)	11	27	0.61	0.65	3.31	0.00	–0.01
QYS	Qinyang, Henan	35.22	112.80	787	12	0.3	4.7	H1(10), **H18(2)**	–	–	–	–	–	–	–
SH	Shahe, Hebei	36.88	114.15	339	9	0	0	H1(9)	9	22	0.51	0.46	2.87	0.00	0.16
SJZ	Shijiazhuang, Hebei	38.07	114.27	200	15	0.13	0.5	H1(14), H3(1)	15	28	0.64	0.55	3.34	0.00	0.17
SLQ	Salaqi, Inner Mongolia	40.60	110.58	1200	8	0	0	H1(8)	8	23	0.48	0.54	2.97	0.13	–0.07
TB	Heshui, Gansu	36.13	108.65	1166	12	0.49	3.7	H10(8), H7(4)	12	27	0.65	0.50	3.54	0.03	0.28*
TC	Tongchuan, Shaanxi	34.92	108.98	623	14	0.26	1	H10(12), **H19(2)**	14	30	0.69	0.53	3.71	0.04	0.27*
WC	Wenchuan, Sichuan	31.48	103.58	1390	10	0.47	1.8	**H13(3), H14(7)**	10	12	0.26	0.26	1.77	0.00	0.04
XX	Xiaoxian, Anhui	34.18	116.90	179	17	0.22	0.8	H1(15), **H4(2)**	17	31	0.56	0.57	3.26	0.13	0.02
XZ	Xinzhou, Shanxi	39.32	113.58	1160	14	0	0	H3(14)	14	28	0.57	0.58	3.33	0.14	0.02
YA	Yanan, Shaanxi	36.58	109.48	1061	13	0.39	10.4	H1(3), **H22(10)**	13	33	0.63	0.62	3.7	0.06	0.07
YJ	Yongji, Shanxi	34.87	110.45	365	9	0.50	5.8	H1(6), **H23(3)**	9	21	0.61	0.61	2.96	0.00	0.05
YT	Yantai, Shandong	37.28	121.73	120	15	0	0	**H24(15)**	15	24	0.55	0.48	2.97	0.00	0.17
ZS	Zishan, Shanxi	35.60	110.97	1300	3	0	0	**H25(3)**	3	8	0.12	0.17	–	–	–0.20

Lat, latitude; Long, longitude; Ele, elevation; *H*_*d*_, haplotype diversity; *A*_*O*_, total observed allele number of six loci; *H*_*O*_, observed heterozygosity; *H*_*E*_, expected heterozygosity; *R*_*S*_, allelic richness; *P*_*AR*_, private allelic richness; *F*_*is*_, inbreeding coefficient.

*significant deviation from zero; –, not genotyped or calculated; haplotypes that are private to only one population are in bold.

### Chloroplast and Nuclear DNA Sequence Analyses

Three chloroplast fragments, *psbJ-petA*, *psbB-psbF*, and *trnL-trnF*, and two species-specific low-copy nuclear genes (*6146* and *38274*) developed using transcriptome data (Fu et al., 2016) following the procedure described by Ye et al. (2017c) were used to detect potential genetic variation in *S. tuberculata* (Supplementary Table 1). The PCRs were performed following the protocol of Ma et al. (2019), except for different annealing temperatures (Supplementary Table 1), and the products were sequenced by Sangon Biotech (Shanghai, China).

cpDNA and nDNA chromatograms were read, edited, and aligned in MEGA 6.0 using the ClustalW algorithm (Tamura et al., 2013) and the alignments were carefully checked manually. For cpDNA, we deleted a region from *psbJ-petA* because it contained complex indels information and was too divergent to be aligned. The three plastid fragments were concatenated after the indels were recoded as single mutations. For nDNA, heterozygote sites were resolved using the coalescent-based Bayesian method of the phase function in DnaSP 5.10 (Librado and Rozas, 2009). Then, for both cpDNA and nDNA, the number of variable sites, number of haplotypes, haplotype diversity (*H*_d_), and nucleotide diversity (π) were calculated in DnaSP. The most parsimonious networks for both plastid and nuclear data were inferred using Network 5.0 (Bandelt et al., 1999). Haplotype distributions were displayed in ArcGis 10.2 (ESRI. Inc.).

Potential population spatial expansion was detected using mismatch distribution analysis and a neutrality test, Tajima’s D (Tajima, 1989) and Fu’s *F*_S_ (Fu, 1997), with default settings for all *S. tuberculata* populations using cpDNA genetic variations in Arlequin 3.5.1.2 (Excoffier and Lischer, 2010). Neither the absolute expansion time nor a Bayesian skyline plot (BSP) (Heled and Drummond, 2008) that can detect changes in effective population size through time were estimated, as no reasonable substitution rate of cpDNA is provided in *S. tuberculata*.

### Nuclear Microsatellites Analyses

Six of the 18 nSSR markers developed for *Speranskia* species (Fu et al., 2016), which produced reproducible, scorable, and polymorphic products, were used to detect genetic variation in *S. tuberculata* (Table 2). The six nSSRs were amplified (Fu et al., 2016) and then genotyped using a 3730xl automated Genetic Analyzer (Applied Biosystems, Foster City, CA, United States). Allele sizes were determined using GENEMAPPER 3.7 (Applied Biosystems). Null alleles were checked using Micro-checker 2.2 (Oosterhout et al., 2004). Linkage disequilibrium was tested using sequential Bonferroni corrections, and the deviation of *F*_is_ (fixation index) from zero was used to test for Hardy-Weinberg equilibrium using FSTAT 2.9.3 (Goudet, 2001).

**TABLE 2:** Measures of genetic diversity and genetic differentiation at six nuclear microsatellite loci in *Speranskia tuberculata*.

**Locus**	***A*_O_**	***H*_O_**	***H*_S_**	***H*_T_**	***F*_ST_**
*9441*	9	0.476	0.505	0.731	0.308
*25439*	9	0.719	0.623	0.745	0.164
*26221*	14	0.461	0.735	0.855	0.141
*10809*	9	0.493	0.568	0.679	0.163
*16226*	6	0.336	0.352	0.497	0.292
*24490*	10	0.489	0.645	0.832	0.224
Mean	9.5	0.496	0.571	0.723	0.215

*A_*O*_, observed allele number; *H*_*O*_, observed heterozygosity; *H*_*S*_, gene diversity; *H*_*T*_, overall gene diversity; *F*_*ST*_, among-population differentiation.*

For all six loci, the genetic diversity index of observed number of alleles (*A*_O_), observed heterozygosity (*H*_O_), gene diversity (*H*_S_), overall gene diversity (*H*_T_), and among-population differentiation (*F*_ST_) were calculated using FSTAT. For all populations, *A*_O_, *H*_O_, and expected heterozygosity (*H*_E_) were calculated in GenAlEx 6.5 (Smouse et al., 2017). The *R*_S_ (allele richness) and *P*_AR_ (private allele richness) in populations with more than eight samples were calculated in hp-rare 1.0 using rarefaction with a sample number of eight (Kalinowski, 2005). To test isolation by distance (IBD), a Mantel test with 10,000 random permutations was performed between the matrix of pairwise *F*_ST_ and that of the natural logarithm of the geographic distances using GenALEx.

The genetic structure was inferred using the Bayesian clustering method in STRUCTURE 2.3 (Falush et al., 2007) with an admixture model and assuming allele frequencies to be correlated among populations. Of the 38 populations sampled, populations DL, JB, and QYS were excluded due to limited sample size or a high proportion of missing data. Ten independent runs were performed for each *K* from 1 to 20 with 1 × 10^5^ burn-in cycles followed by 1 × 10^6^ MCMC cycles. Potential clusters (*K*) were determined by *LnP(D)*, the change in log-likelihood of the data for each run (Pritchard et al., 2000), and Δ*K*, the second-order rate of change of *LnP(D)* between successive *K-*values (Evanno et al., 2005).

### Ecological Niche Modeling

To explore the historical distribution range shifts of *S. tuberculata*, its potential distribution at present and during the LGM were modeled using MAXENT (Phillips et al., 2006). A total of 94 occurrence points were collected from our field records (35) and herbarium records (59). Eight low-correlated (*r* < 0.75) climate variables (Supplementary Table 2, available at^1^) with 2.5 arc-min resolution were used to model the niche at present (Hijmans et al., 2005). The established model was projected onto the LGM climatic conditions reconstructed using the Community Climate System Model (CCSM) (Collins et al., 2006) at 2.5 arc-min resolution. We used 80% of the species records for training and 20% for model testing with 20 replicates. The accuracy of the model’s performance was evaluated based on the area under the receiver operating characteristic curve (AUC) (Fawcett, 2006).

## Results

### cpDNA Analyses

**FIGURE 1:**
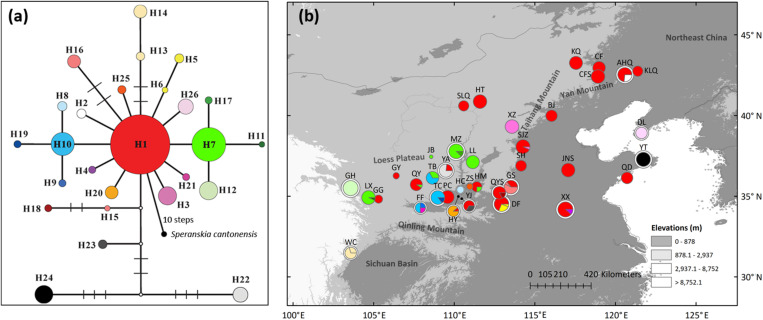
Most parsimonious network **(a)** and geographic distribution **(b)** of 26 chloroplast DNA haplotypes of *Speranskia tuberculata*. In **(a)**, the small white dots without labels in the network represent missing alleles; *S. cantonensis* served as outgroup. Private haplotypes distributed only in one population are shown in the same color (H13 and H14 in WC, H15 and H16 in GS). In **(b)**, Populations that have private haplotypes are labeled with white outer ring. All circle sizes are proportional to sample sizes.

**FIGURE 2:**
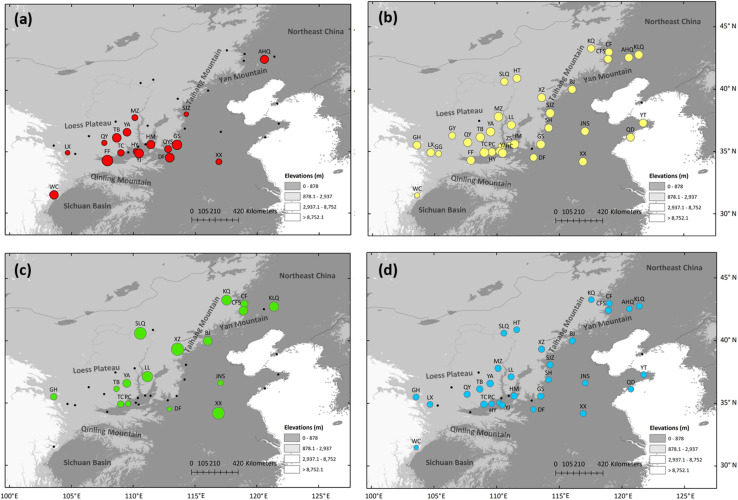
Genetic diversity estimated via haplotype diversity (*H*_d_) of chloroplast DNA **(a)**, expected heterozygosity (*H*_E_, **b**), private allelic richness (*P*_AR_, **c**), and allelic richness (*R*_S_, **d**) of nuclear microsatellites of *Speranskia tuberculata*. Black dots indicate populations with zero or non-calculated genetic diversity.

Overall, 34 variable sites including 15 indels were detected in 423 *S. tuberculata* individuals, and 26 haplotypes were identified with a total of 2,600 bp using three chloroplast fragments (Supplementary Table 3). The most parsimonious network showed that most haplotypes were closely related, except H22 and H24. The most abundant and centrally located haplotype H1 showed the closest relationship with outgroup *S. cantonensis* (Figure 1a). Haplotype H1 is widely distributed across the entire range of *S. tuberculata*, especially outside of the CLP (Figure 1b). Two distantly related haplotypes (H15 and H16) were both distributed in population GS, and three relatively closely related haplotypes (H22–H24) showed disjunct distributions between the CLP (YA and YJ population) and coastal regions (YT population) (Figure 1b). Populations that have private haplotypes were mainly distributed in the CLP except two found in the coastal region (DL and YT population) (Figure 1b). Populations with high haplotype diversity were mainly located in the southern CLP and adjacent regions except the AHQ population in northeastern China (Figure 2a).

Both mismatch analysis (sum of squares deviation = 0.01, *P* = 0.327, Harpending’s Raggedness index = 0.04, *P* = 0.845, Supplementary Figure 1) and the neutrality tests (Tajima’s *D* = −1.72, *P* = 0.01, Fu’s *F*_S_ = −12.29, *P* = 0) indicated significant spatial expansion for all populations.

### nDNA Analyses

**FIGURE 3:**
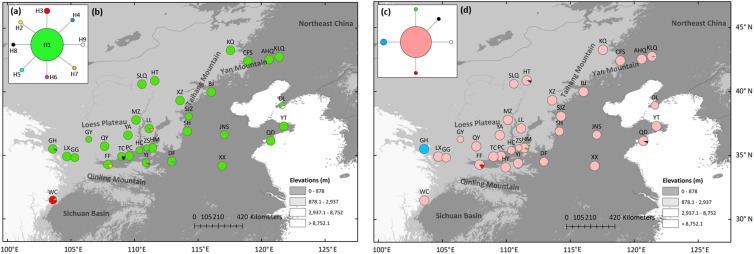
Most parsimonious network **(a,c)** and geographic distribution **(b,d)** of *Speranskia tuberculata* haplotypes of low-copy nuclear genes of *38274* **(a,b)** and *6146* **(c,d)**, respectively.

Low genetic variation was found in both nuclear loci (*6146* and *38274*, Supplementary Table 4). In locus *38274*, eight variable sites in 405 bp of 181 individuals resulted in nine haplotypes. Haplotypes H2–H9 were all one mutation away from the centrally located haplotype H1 (Figure 3a). Private haplotypes were distributed in populations south of the Qinling Mountains (WC population), the southern CLP (GH, FF, TC, LL, and YJ population), and coastal regions (DL population) (Figure 3b). In locus *6146*, six haplotypes were detected through five variable sites along 294 bp in 184 individuals. H1 was separated from H2–H5 by only one mutation site (Figure 3c). H1 was widely distributed, and three populations that have private haplotypes (H3 in GH, H2 in FF, and H4 in the HM population) were distributed in the southern CLP (Figure 3d).

### nSSRs Analyses

Null alleles were not found in the six nSSR loci, and no significant genotypic disequilibrium was observed among the 15 locus pairs in any populations. Several populations had a fixation index (*F*_is_) that is significantly deviated from zero, suggesting that these populations are not under Hardy-Weinberg equilibrium (Table 1). The genetic diversity indices of the 35 sampled populations and six loci are listed in Tables 1 and 2, respectively. Within-population genetic diversities, *H*_E_, *P*_AR_, and *R*_S_, are all uniformly distributed (Figures 2b–d). A Mantel test (*r* = 0.171, *P* = 0.037) indicated a significant correlation between genetic differentiation and geographic distance among all sampled populations.

**FIGURE 4:**
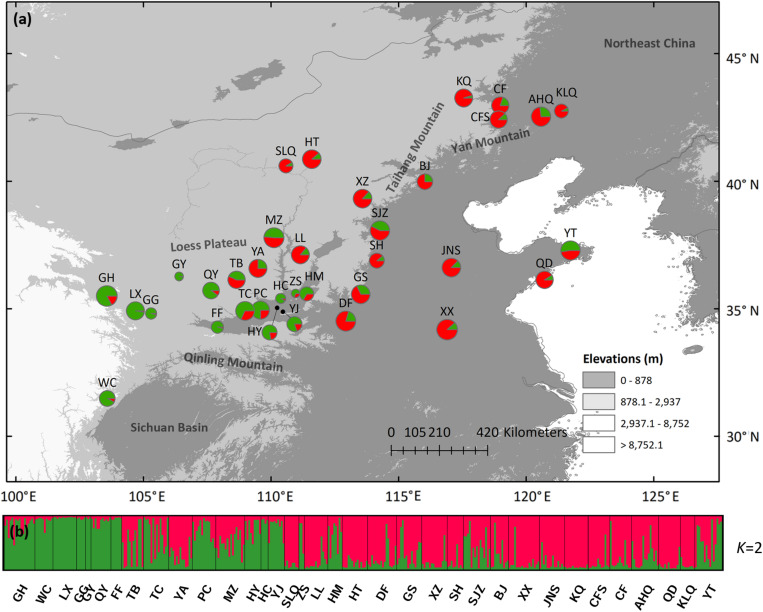
**(a)** Color-coded grouping of the 35 *Speranskia tuberculata* populations according to the most likely *K* = 2 inferred from STRUCTURE analysis. **(b)** Histogram of the STRUCTURE assignment test at *K* = 2.

Using STRUCTURE, the highest Δ*K* is observed at *K* = 2, while the *LnP (D)* gradually increased from *K* = 2 to *K* = 11 (Supplementary Figure 2). We chose *K* = 2 as the most possible clustering (Figure 4a), and the histogram of the STRUCTURE assignment test for all populations from *K* = 2 is shown in Figure 4b. The western green cluster is mainly distributed in the CLP and adjacent regions, while the eastern red cluster is in the eastern part of the present *S. tuberculata* distribution range (Figure 4a). Pure western cluster populations were observed in many populations, while pure eastern populations were barely found.

### Ecological Niche Modeling

**FIGURE 5:**
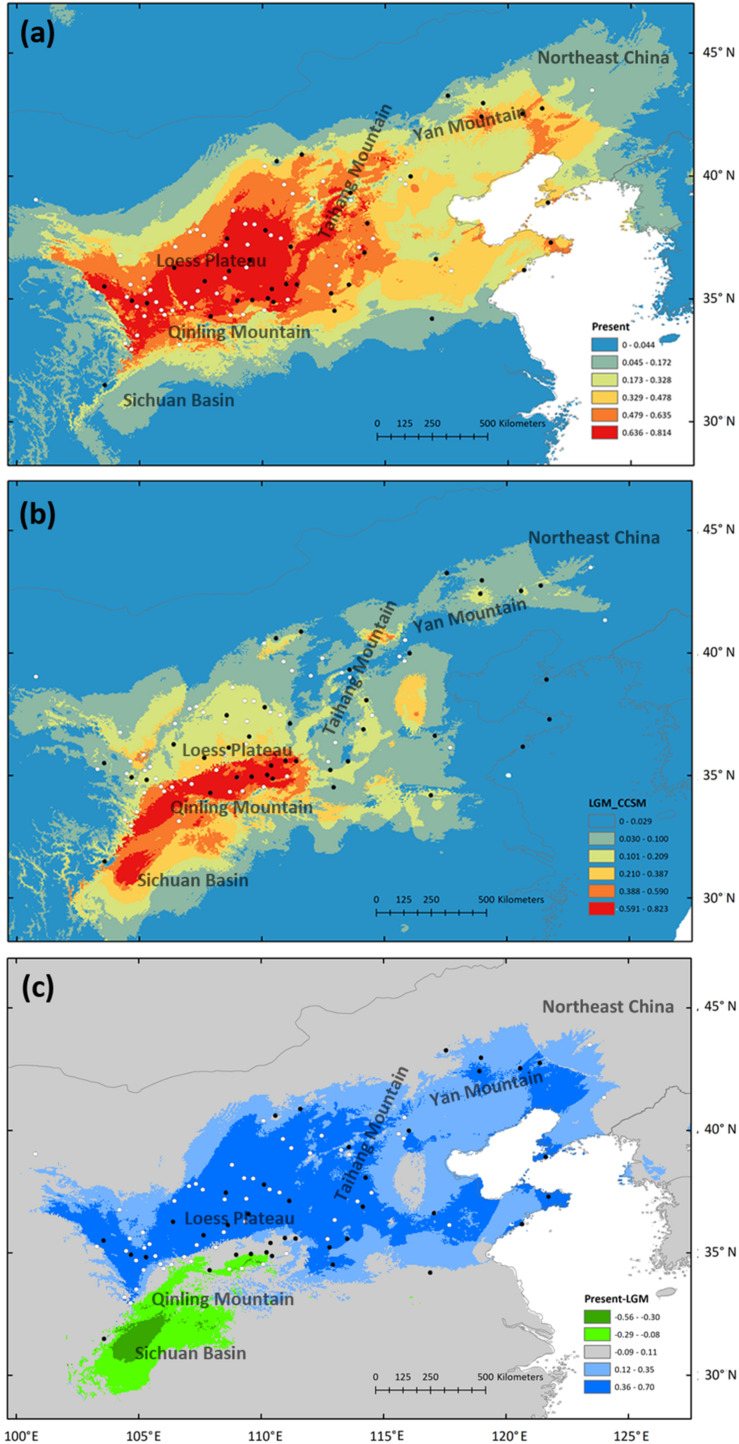
Potential species distribution of *Speranskia tuberculata* estimated by ecological niche modeling at present **(a)** and during the last glacial maximum (LGM) using the CCSM3 model **(b)**. Absolute changes in habitat suitability are compared between present and LGM **(c)**; Blue represents suitability gains and green represents losses in suitability. Black dots represent locations of sampled populations, while white dots represent occurrence points collected from herbarium records.

High ROC values (0.968 ± 0.012) indicate good accuracy of model predictions. At present, *S. tuberculata* is distributed mainly in the CLP and adjacent regions with extensions from Taihang-Yan mountains to northeastern China and from the North China Plain to the coastal region (Figure 5a). During the LGM, the most suitable habitat is predicted to have been located in the southern CLP and the Qinling Mountains with extension to the Sichuan Basin (Figure 5b). Comparison of absolute changes in habitat suitability showed that the northern distribution of *S. tuberculata* was reached after the LGM from the southern CLP and that the species has experienced habitat loss in the Sichuan Basin (Figure 5c).

## Discussion

### Southern Macrorefugium and Multiple Distant Microrefugia

In the present study, the phylogeography history of *S. tuberculata* was investigated using multiple genetic markers and potential habitat modeling. Chloroplast and nuclear DNA as well as ENM consistently support the hypothesis that *S. tuberculata* survived the unsuitable climate in the macrorefugium in the southern CLP and in multiple distant microrefugia. In contrast, nSSRs are ambiguous in inferring refugia and postglacial demographic history.

In chloroplast and nuclear sequences, populations with high haplotype diversity and private haplotypes are mainly distributed in the southern CLP, indicating that the macrorefugium of *S. tuberculata* is located there (Hewitt, 1996, 2004). Disjointly distributed closely related or co-distributed distant chloroplast haplotypes are evidence of population loss during past climate changes (Qiu et al., 2011), likely due to the cold and dry climate during the LGM. The mismatch distribution and the neutrality tests of chloroplast sequences support spatial expansion, which results in a wide distribution of haplotype H1 (Figure 1b). ENM also shows that most of the present *S. tuberculata* distribution north of the Qinling Mountains has been gained after the LGM (Figure 5). Post-glacial colonizations to the eastern part of the present distribution are likely through the Taihang-Yan Mountains and the North China Plain. Private chloroplast or nuclear haplotypes in northeastern China (KLQ and AHQ population) or in coastal regions (DL and YT population) may indicate multiple restricted microrefugia (Provan and Bennett, 2008). Therefore, *S. tuberculata* is likely to have persisted in the southern CLP macrorefugium and in multiple microrefugia in northeastern China and the coastal region (Ye et al., 2017b).

Seemingly, the nSSRs data tell a different story as two different scenarios can both generate genetic patterns displayed by nSSRs. First, expansions from an eastern and a western macrorefugium can result in genetically mixed populations in the central contact zone (Figure 4), similar to some widely distributed species (e.g., *Acer mono* and *Juglans* spp.) that show a genetic mixture in northern China (Guo et al., 2014; Bai et al., 2016). Such populations in contact zones are expected to have higher genetic diversity (Petit et al., 2003). However, this is not the case for *S. tuberculata* (Figures 3b–d), additionally pure populations belonging to the eastern cluster are barely found. Besides, uniformly distributed genetic diversity is probably attributed to massive gene flow via wind-dispersed pollen, which can reduce the surfing effect during spatial expansion and bring the majority of genetic composition to the expansion front (Wang et al., 2016). This scenario could explain that genetic structure changes gradually from west to east in agreement with a significant IBS pattern (Figure 4). Thus, nSSR a data are compatible with the southern macrorefugium scenario.

### The Significance of the CLP in Preserving Biodiversity

In previous phylogeographic studies in northern China, detected refugia were mainly located in mountain regions, such as the Qingling Mountains (Tian et al., 2009) or the Yan-Taihang Mountains (Zeng et al., 2011), but not in the CLP. In some other species mainly distributed in arid northwestern China, the CLP acts as a dispersal corridor for colonization (*Lagochilus ilicifolius*, Meng and Zhang, 2011; *Ribes meyeri*, Xie and Zhang, 2013). In this study, using the drought-enduring herb *S. tuberculata* as a case, multiple genetic data and potential habitat modeling uncovered the role of the CLP as a macrorefugium, indicating its importance in preserving biodiversity in northern China.

The CLP is mainly influenced by the Asian winter monsoon, which brings dust from the deserts of the Asian interior. The initial eolian dust deposits could dates back to the Eocene-Oligocene (Licht et al., 2014; Clift et al., 2015). Such deposits experienced several intensifications up to the Quaternary (Wang et al., 2007). Precipitation, which is higher in the southeast and lower in the northwest, has a direct influence on the vegetation of the CLP as it is located in the dry interior of Asia (Zhang et al., 2012), and this gradient was also present in the interglacial periods (Jiang and Ding, 2005). During the LGM, as the CLP is located in the marginal zone of the Asian summer monsoon, the climate is prone to be the same compared to prominent north-south and west-east gradients (warmer and wetter conditions in the south/east) during the interglacial period (Jiang et al., 2014). Higher precipitation is needed to develop wood forest while lower precipitation is needed for glass forest (Lv et al., 1999), and herbaceous species are thought to be more likely to find refugia in the CLP than tree species that survived the LGM in mountain regions (Tian et al., 2009; Zeng et al., 2011). In the CLP flora, many genera that are adapted to arid environments (Wang, 1999), such as *Caragana*, *Ephedra*, and *Tamarix*, are highly diversified, and majority of the CLP endemic species show tolerance to aridity (Zhang et al., 2002). In addition to the herb *S. tuberculata*, other drought-enduring species are also likely to have persisted in the CLP macrorefugium and to have experienced post-glacial expansions.

The CLP contributes not only to the biodiversity of northern China, but also to other regions in East Asia. First, Meng et al. (2015) have suggested the CLP to be an important refugium for the dry-adapted flora of northwestern China. Furthermore, disjoint distribution of same haplotype between CLP and northeastern China may result from long distance dispersal from the CLP (Tian et al., 2009; Song et al., 2020). Finally, haplotypes of *S. tuberculata* in the Sichuan Basin (H13–14) are derived from the ancestral haplotype (H1), indicating the CLP may be the source of genotypes in southwestern China.

## Conclusion

The present phylogeographic work is an important supplement to previous studies in East Asia, which are much more numerous for northwestern China (Meng et al., 2015), subtropical China (Ye et al., 2017a), northeastern China (Ye et al., 2017b) or southwestern China (Liu et al., 2017). However, further phylogeographic studies on various species are still needed to depict a comprehensive response of the flora from northern China to Quaternary climate changes (Zhang et al., 2002).

## Data Availability Statement

The datasets presented in this study can be found in online repositories. The names of the repository/repositories and accession number(s) can be found in the article/Supplementary Material.

## Author Contributions

J-WY and BT conceived the ideas. H-YW, M-JF, and PZ contributed to the sampling and the data analyses. J-WY and BT wrote the manuscript. All authors have read and approved the final manuscript.

## Conflict of Interest

The authors declare that the research was conducted in the absence of any commercial or financial relationships that could be construed as a potential conflict of interest.

## Footnotes

1 http://www.worldclim.org/

## Supplementary Material

Supplementary Figure 1 Mismatch distribution of simulation under spatial expansion assuming constant deme size for all *Speranskia tuberculata* populations.

Supplementary Figure 2 Delta-*K* **(A)** and Ln*P*(*D*) **(B)** in STRUCTURE analysis on all *Speranskia tuberculata* populations are shown for predefined group number *K* = 1–20. Standard deviations of Ln*P* (*D*) obtained from 10 independent runs for each predefined group size are also shown.
