# Supplementary figures and images for: Insights Into the Significance of the Chinense Loess Plateau for Preserving Biodiversity From the Phylogeography of Speranskia tuberculata (Euphorbiaceae)
Source: Front Plant Sci. 2021 Feb 4;12:604251. doi: 10.3389/fpls.2021.604251 (PMC7889603; doi:10.3389/fpls.2021.604251)

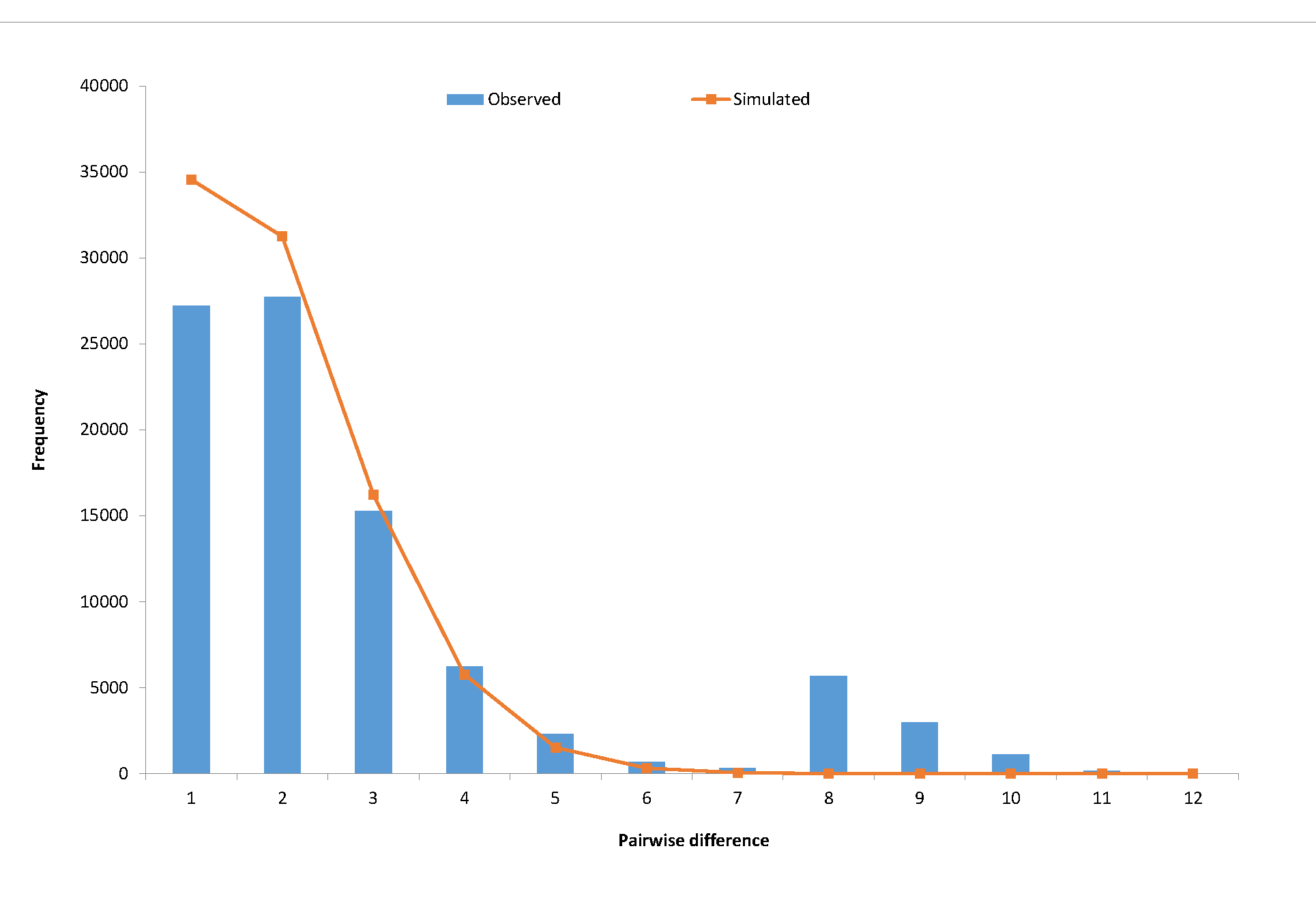

Supplement: Supplementary Figure 1 — Mismatch distribution of simulation under spatial expansion assuming constant deme size for all Speranskia tuberculata populations. [file Image_1.TIF]

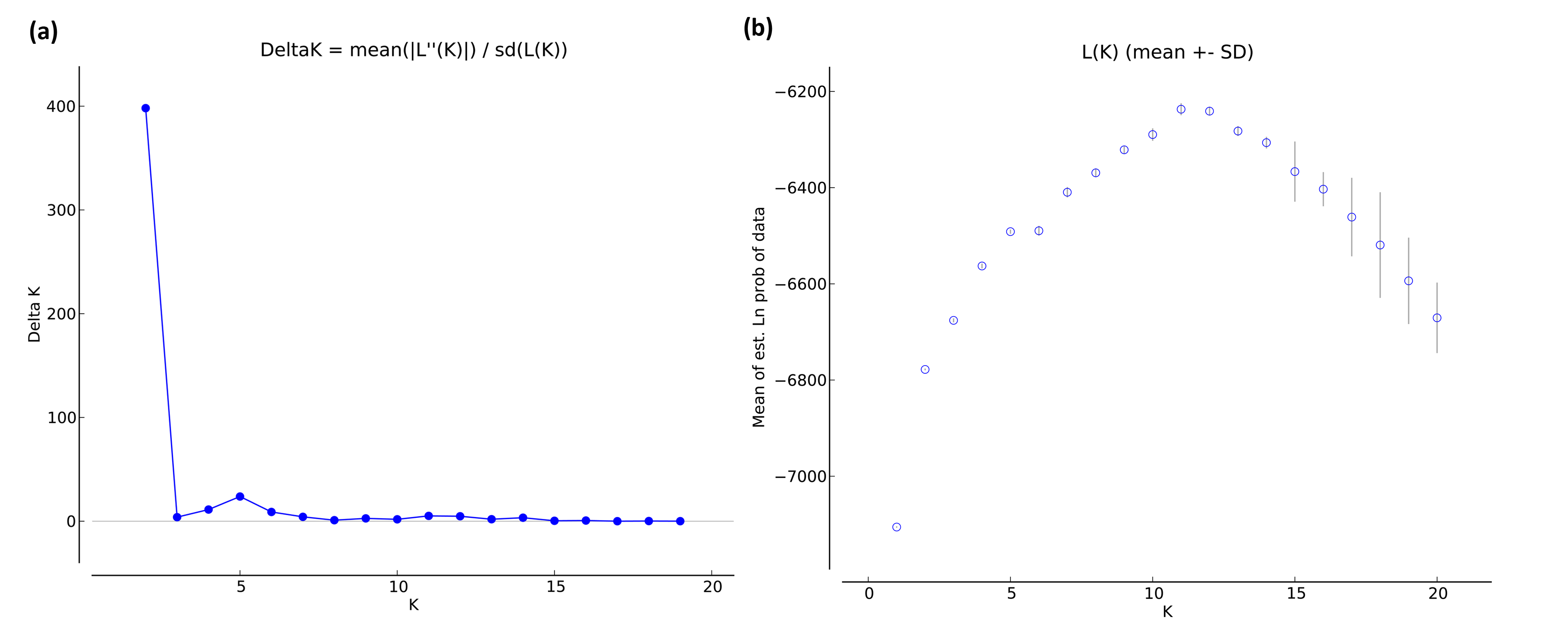

Supplement: Supplementary Figure 2 — Delta-K (A) and LnP(D) (B) in STRUCTURE analysis on all Speranskia tuberculata populations are shown for predefined group number K = 1–20. Standard deviations of LnP (D) obtained from 10 independent runs for each predefined group size are also shown. [file Image_2.TIF]
