# Supplementary material for: Insights Into the Significance of the Chinense Loess Plateau for Preserving Biodiversity From the Phylogeography of Speranskia tuberculata (Euphorbiaceae)
Source: Front Plant Sci. 2021 Feb 4;12:604251. doi: 10.3389/fpls.2021.604251 (PMC7889603; doi:10.3389/fpls.2021.604251)
Supplement: Supplementary file 3 [file Table_1.DOCX]

| **Supplementary Table S1** Primers used for chloroplast and nuclear DNA sequencing in *Speranskia tuberculata*. | | | | |
| --- | --- | --- | --- | --- |
| Fragment/locus | Length (bp) | Annealing temperature (°C) | Primer | Primer sequence |
| *psbB-psbF* | 852 | 56 | F | GTTTACTTTTGGGCATGCTTCG |
|  |  |  | R | CGCAGTTCGTCTTGGACCAG |
| *trnL-trnF* | 969 | 56 | F | CGAAATCGGTAGACGCTACG |
|  |  |  | R | ATTTGAACTGGTGACACGAG |
| *psbJ-petA* | 779 | 56 | F | ATAGGTACTGTARCYGGTATT |
|  |  |  | R | AACARTTYGARAAGGTTCAATT |
| *6146* | 294 | 59 | F | GTCGGATACAAGAACGCGTC |
|  |  |  | R | GCGTTTACAGCGATCAAGGT |
| *38274* | 405 | 59 | F | AGAGGTGCCCCATCATTGAA |
|  |  |  | R | ACTGCTCTCAATCCTTGGCT |
