# Supplementary material for: Insights Into the Significance of the Chinense Loess Plateau for Preserving Biodiversity From the Phylogeography of Speranskia tuberculata (Euphorbiaceae)
Source: Front Plant Sci. 2021 Feb 4;12:604251. doi: 10.3389/fpls.2021.604251 (PMC7889603; doi:10.3389/fpls.2021.604251)
Supplement: Supplementary file 4 [file Table_2.DOCX]

| **Supplementary Table S2** Eight selected bioclimatic variables with low correlations (r < 0.75) used in ecological niche modelling for *Speranskia tuberculata*. | |
| --- | --- |
| Variable | Description |
| BIO1 | Annual mean temperature |
| BIO2 | Mean diurnal range (mean of monthly (max temp - min temp)) |
| BIO3 | Isothermality (BIO2/BIO7*) (× 100) |
| BIO4 | Temperature seasonality (SD × 100) |
| BIO12 | Annual precipitation |
| BIO13 | Precipitation of wettest month |
| BIO14 | Precipitation of driest month |
| BIO15 | Precipitation seasonality (coefficient of variation) |
| *Bio7, annual temperature annual range (Bio5 − Bio6); Bio5, Max temperature of warmest month, Bio6, Minimum temperature of the coldest month | |
