# Supplementary material for: Insights Into the Significance of the Chinense Loess Plateau for Preserving Biodiversity From the Phylogeography of Speranskia tuberculata (Euphorbiaceae)
Source: Front Plant Sci. 2021 Feb 4;12:604251. doi: 10.3389/fpls.2021.604251 (PMC7889603; doi:10.3389/fpls.2021.604251)
Supplement: Supplementary file 5 [file Table_3.DOCX]

| **Supplementary Table S3** Haplotypes derived from four chloroplast DNA fragments in *Speranskia tuberculata*: *psbB-psbF* (positions 1–852), *psbJ-petA* (positions 853-1631) and *trnL-trnF* (positions 1632–2600). | | | | | | | | | | | | | | | | | | | | | | | | | | | | | | | | | | | | | |
| --- | --- | --- | --- | --- | --- | --- | --- | --- | --- | --- | --- | --- | --- | --- | --- | --- | --- | --- | --- | --- | --- | --- | --- | --- | --- | --- | --- | --- | --- | --- | --- | --- | --- | --- | --- | --- | --- |
| Haplotype | n | 90 | 132 | 187 | 203 | 223 | 225 | 304 | 466 | 493 | 683 | 699 |  | 1048 | 1049 | 1159 | 1231 | 1257 | 1271 | 1292 | 1297 | 1397 | 1434 | 1562 |  | 1739 | 1748 | 1804 | 1845 | 1929 | 1933 | 2051 | 2223 | 2237 | 2360 | 2505 | 2525 |
|  |  | A | T | C | C | - | - | 3 | C | - | - | - |  | - | C | C | - | G | - | T | G | T | - | - |  | G | T | G | A | T | A | - | - | - | A | A | - |
| H1 | 225 | — | — | — | — | - | - | — | — | - | - | - |  | - | — | — | - | — | - | — | — | — | - | - |  | — | — | — | — | — | — | - | - | - | — | — | - |
| H2 | 4 | — | — | — | — | - | - | — | — | - | - | - |  | - | — | — | - | — | - | — | — | — | - | - |  | — | — | — | — | — | — | - | 13 | - | — | — | - |
| H3 | 15 | — | — | — | — | - | - | — | — | - | - | - |  | - | — | — | - | — | - | — | — | — | - | - |  | T | — | — | — | — | — | - | - | - | — | — | - |
| H4 | 2 | C | — | — | — | - | - | — | — | - | - | - |  | - | — | — | - | — | - | — | — | — | - | - |  | — | — | — | — | — | — | - | - | - | — | — | - |
| H5 | 4 | — | C | — | — | - | - | — | — | - | - | - |  | - | — | A | - | — | - | — | — | — | - | - |  | — | — | — | — | — | — | - | - | - | — | — | - |
| H6 | 1 | — | — | — | — | - | - | — | — | - | - | - |  | - | — | A | - | — | - | — | — | — | - | - |  | — | — | — | — | — | — | - | - | - | — | — | - |
| H7 | 49 | — | — | — | — | - | - | — | A | - | - | - |  | - | — | — | - | — | - | — | — | — | - | - |  | — | — | — | — | — | — | - | - | - | — | — | - |
| H8 | 4 | — | — | — | — | - | - | — | — | - | - | - |  | A | — | — | - | — | - | — | — | — | - | 11 |  | — | — | — | — | — | — | - | - | - | — | — | - |
| H9 | 2 | — | — | — | — | - | - | — | — | - | - | - |  | - | — | — | - | — | - | — | — | — | - | 11 |  | — | — | — | — | — | C | - | - | - | — | — | - |
| H10 | 24 | — | — | — | — | - | - | — | — | - | - | - |  | - | — | — | - | — | - | — | — | — | - | 11 |  | — | — | — | — | — | — | - | - | - | — | — | - |
| H11 | 1 | — | — | — | — | - | - | — | A | - | - | - |  | - | — | — | - | — | - | — | — | — | - | - |  | — | — | — | — | — | — | - | - | - | — | G | - |
| H12 | 17 | — | — | — | — | 1 | - | — | A | - | - | - |  | - | — | — | - | — | - | — | — | — | - | - |  | — | — | — | — | — | — | - | - | - | — | — | - |
| H13 | 3 | — | — | — | A | - | - | — | — | - | - | - |  | - | — | — | - | — | - | — | — | — | - | - |  | — | — | — | — | — | — | 12 | - | - | — | — | - |
| H14 | 7 | — | — | — | A | - | - | — | — | - | 5 | - |  | - | — | — | - | — | - | — | — | — | - | - |  | — | — | — | — | — | — | 12 | - | - | — | — | - |
| H15 | 1 | — | — | — | — | - | - | — | — | - | - | 6 |  | - | — | — | - | — | 8 | — | — | — | - | - |  | — | — | — | — | — | — | - | - | - | — | — | - |
| H16 | 8 | — | — | — | — | - | 2 | — | — | - | - | - |  | - | — | — | - | — | - | — | — | — | - | - |  | — | — | — | G | — | — | - | - | - | — | — | - |
| H17 | 2 | — | — | — | — | - | - | — | A | - | - | - |  | - | — | — | - | — | - | — | — | — | - | - |  | — | A | — | — | — | — | - | - | - | — | — | - |
| H18 | 2 | — | — | — | — | - | - | - | — | - | - | 6 |  | - | — | — | - | — | 8 | — | — | — | - | - |  | — | — | A | — | — | — | - | - | - | — | — | - |
| H19 | 2 | — | — | — | — | - | - | — | — | - | - | - |  | - | — | — | - | — | - | C | — | — | - | 11 |  | — | — | — | — | — | — | - | - | - | — | — | - |
| H20 | 8 | — | A | — | — | - | - | — | — | - | - | - |  | - | — | — | - | — | - | — | — | — | - | - |  | — | — | — | — | — | — | - | - | - | — | — | - |
| H21 | 2 | — | — | — | — | - | - | — | — | - | - | - |  | - | — | — | - | — | - | — | — | — | - | - |  | — | — | — | — | — | — | - | - | - | G | — | - |
| H22 | 10 | — | — | T | — | - | - | — | — | - | - | - |  | - | — | — | 7 | A | 8 | — | — | — | 10 | - |  | — | G | — | — | — | — | - | - | - | — | — | 15 |
| H23 | 3 | — | — | — | — | - | - | — | — | - | - | - |  | - | — | — | - | A | 8 | — | T | — | - | - |  | — | — | — | — | — | — | - | - | - | — | — | - |
| H24 | 15 | — | — | — | — | - | - | — | — | - | - | - |  | - | A | — | 7 | A | 8 | — | — | 9 | - | - |  | — | — | — | — | A | — | - | - | - | — | — | 15 |
| H25 | 3 | — | — | — | — | - | - | — | — | 4 | - | - |  | - | — | — | - | — | - | — | — | — | - | - |  | — | — | — | — | — | — | - | - | - | — | — | - |
| H26 | 10 | — | — | — | — | - | - | — | — | - | - | - |  | - | — | — | - | — | - | — | — | — | - | - |  | — | — | — | — | — | — | - | - | 14 | — | — | - |
| -, indel; 1, ATTATTA; 2, TTATTATT 3, GAATCTTG; 4, GTTATCTTAATT; 5, TATATAAA; 6, CCTTTTTTTGATA; 7, TATATTTT; 8, TTTTTC; 9, TTCTTTTTTTG; 10, TTTTTTAC; 11, CATTATTATTTTGACACA; 12, AGTTTGATAGATAACTTATTAATCG; 13, TTTTA; 14, TTCTATCTT; 15, CTTTTTTTTTTAATTGA. | | | | | | | | | | | | | | | | | | | | | | | | | | | | | | | | | | | | | |
