# Supplementary material for: Insights Into the Significance of the Chinense Loess Plateau for Preserving Biodiversity From the Phylogeography of Speranskia tuberculata (Euphorbiaceae)
Source: Front Plant Sci. 2021 Feb 4;12:604251. doi: 10.3389/fpls.2021.604251 (PMC7889603; doi:10.3389/fpls.2021.604251)
Supplement: Supplementary file 6 [file Table_4.DOCX]

| **Supplementary Table S4** Sample size and haplotype distribution of two low-copy nuclear genes in *Speranskia tuberculata*. | | | | | |
| --- | --- | --- | --- | --- | --- |
| Population | *6146* | |  | *38274* | |
|  | sample size | Haplotype (n) |  | sample size | Haplotype (n) |
| AHQ | 12 | H1(12) |  | 12 | H1(12) |
| BJ | 12 | H1(12) |  | 12 | H1(12) |
| CFS | 12 | H1(12) |  | 12 | H1(12) |
| DF | 10 | H1(10) |  | 12 | H1(11)H5(1) |
| DL | 8 | H1(8) |  | 6 | H1(4)H9(2) |
| FF | 12 | H1(10)H2(2) |  | 12 | H1(10)H2(2) |
| GG | 10 | H1(10) |  | 10 | H1(10) |
| GH | 12 | H3(12) |  | 12 | H1(11)H4(1) |
| GY | 6 | H1(6) |  | 6 | H1(6) |
| HC | 8 | H1(8) |  | 8 | H1(8) |
| HM | 12 | H1(11)H4(1) |  | 12 | H1(12) |
| HT | 12 | H1(11)H5(1) |  | 12 | H1(12) |
| HY | 12 | H1(12) |  | - | - |
| JNS | 10 | H1(10) |  | 10 | H1(10) |
| KLQ | 12 | H1(11)H6(1) |  | 12 | H1(12) |
| KQ | 12 | H1(12) |  | 12 | H1(12) |
| LL | 12 | H1(12) |  | 12 | H1(10)H7(2) |
| LX | 12 | H1(12) |  | 12 | H1(12) |
| MZ | 12 | H1(12) |  | 12 | H1(12) |
| PC | 12 | H1(12) |  | 12 | H1(12) |
| QD | 12 | H1(11)H5(1) |  | 12 | H1(12) |
| QY | 12 | H1(12) |  | 12 | H1(12) |
| SH | 10 | H1(10) |  | 12 | H1(12) |
| SJZ | 12 | H1(12) |  | 8 | H1(8) |
| SLQ | 10 | H1(10) |  | 12 | H1(12) |
| TC | 12 | H1(12) |  | 10 | H1(8) H8(2) |
| WC | 12 | H1(12) |  | 10 | H1(1) H3(9) |
| XX | 12 | H1(12) |  | 12 | H1(12) |
| XZ | 12 | H1(12) |  | 12 | H1(12) |
| YA | 12 | H1(12) |  | 12 | H1(12) |
| YJ | 12 | H1(12) |  | 10 | H1(9) H6(1) |
| YT | 12 | H1(12) |  | 12 | H1(12) |
| ZS | 6 | H1(6) |  | 6 | H1(6) |

-, not sequenced.
